# Mid-term results of the floating stitch for systolic anterior motion in hypertrophic obstructive cardiomyopathy

**DOI:** 10.1007/s11748-025-02167-6

**Published:** 2025-06-12

**Authors:** Tomonari Uemura, Akihiko Usui, Yoshiyuki Tokuda, Yuji Narita, Masato Mutsuga

**Affiliations:** 1 Department of Cardiac Surgery, Nagoya University Graduate School of Medicine, 65 Tsurumai-Cho, Showa-Ku, Nagoya, Aichi 466-8560 Japan; 2 Department of Cardiovascular Surgery, Fujita Health University Okazaki Medical Center, Okazaki, Aichi Japan

**Keywords:** Systolic anterior motion, Hypertrophic obstructive cardiomyopathy, Mitral valve plasty

## Abstract

**Objective:**

Systolic anterior motion (SAM) is an important factor for hypertrophic obstructive cardiomyopathy (HOCM) patients with a hypertrophic interventricular septum. We developed the ‘floating stitch technique’ to relieve SAM and have used it since 2017. The mid-term results of the floating stitch technique are reported.

**Methods:**

Ten consecutive HOCM patients (5 male, mean age 65.6 years) who underwent septal myectomy and the floating stitch technique from 2017 to 2022 were included. All patients underwent preoperative, pre-discharge, and annual follow-up echocardiographic evaluations. The median postoperative observation period was 3.5 (range 1.2–6.6) years.

**Results:**

There were no cases of cutting or elongation of the floating stitch during the follow-up period. The median mitral valve area (MVA) was 2.9 [interquartile range (IQR) 2.6–3.1] cm^2^ before surgery, 2.6 (IQR 2.2–2.7) cm^2^ before discharge, and 2.6 (IQR 2.2–2.8) cm^2^ at the latest follow-up. There were no cases of mitral stenosis clinically. All cases showed a significant decrease in the left ventricular outflow tract pressure gradient after surgery, but one case required re-operation due to recurrent obstruction at the mid-cardiac position. SAM did not recur in any cases, and all patients were in NYHA class 1 at the latest follow-up.

**Conclusions:**

The floating stitch technique showed an excellent SAM-suppression effect and durability. MVA decreased about 10% following the floating stitch technique, but sufficient area was secured without functional mitral stenosis. The combination of septal myectomy and floating stitch technique is a simple and reproducible procedure for HOCM, especially with severe SAM.

**Supplementary Information:**

The online version contains supplementary material available at 10.1007/s11748-025-02167-6.

## Introduction

Septal myectomy is an established surgical procedure for left ventricular outflow tract (LVOT) obstruction. This procedure reduces LVOT obstruction by widening the outflow tract, which decreases blood flow velocity and the pressure gradient. In addition, it can improve mitral valve regurgitation (MR) to some extent [1]. However, septal myectomy alone may be insufficient, particularly in patients with significant preoperative SAM. When mitral valve abnormalities are detected preoperatively or intraoperatively, concomitant mitral valve intervention along with myectomy is necessary to obtain optimal surgical outcomes [2].

Several surgical techniques involving the mitral valve leaflet and sub-valvular apparatus have been reported. We developed the ‘floating stitch technique’ and have used it since 2017 to prevent postoperative SAM after HOCM surgery [3]. This technique is particularly useful for cases with severe preoperative SAM.

### Objective

In this study, the objective was to investigate the mid-term outcomes of HOCM patients who underwent surgery with our floating stitch technique.

## Patients and methods

The study was approved by the Research Ethics Committee of Nagoya University Hospital (IRB number: 2019–0179; date of approval: 2019/08/23). The need to obtain informed consent was waived based on the retrospective nature of the investigation.

### Study population and clinical presentation

Ten consecutive patients with HOCM who underwent septal myectomy in addition to floating stitch technique for HOCM from 2017 to 2022 were included in this study. During the study period, septal myectomy was performed in 29 HOCM cases, septal myectomy with mitral valve procedures was performed in 20 cases, and the floating stitch technique was applied in 10 cases. The floating stitch technique was principally applied for cases with MR due to severe SAM. Initially, this technique was performed for moderate or greater MR due to SAM, but from around 2020, we began to apply it to mild MR cases as well. This is because we found that the floating stitch does not compromise mitral valve function and is easy to perform. Five patients were male, and the patients’ mean age was 65.6 years. On preoperative electrocardiography, nine patients were in sinus rhythm, and one was in atrial fibrillation. With appropriate drug therapy, the patients’ NYHA classifications were class 2 in four patients and class 3 in six patients. The LVOT pressure gradient, LV function, degree of mitral regurgitation, interventricular septal thickness (IVST), and distance from the coaptation point to the septum (C-sept) were evaluated by transthoracic echocardiography. C-sept was measured as the shortest distance from the coaptation point to the interventricular septum in the apical left ventricular long-axis view during mid-systole. The peak LVOT pressure range (P) was estimated by the modified Bernoulli equation (P = 4V^2^) from Doppler echocardiographic velocity (V) assessment.

Mitral valve opening area was evaluated by determining MVA using the estimation equation from pressure half-time (PHT) (see below). Preoperative patient background characteristics are shown in Table 1.

**Table 1:** Clinical and echocardiographic variables of the 10 patients

Variable	Value
Age, y	63.3 ± 11.2
Male, *n* (%)	5 (50)
IVST, mm	25.4 [22.1–30.9]
EF, %	64.2 [57.7–69.8]
LVOT gradient, mmHg	90.5 [63.2–149]
MR grade (0–4)	
0–1	1 (10)
2	5 (50)
3–4	4 (40)
C-sept, mm	3.15 [2.53–4.35]
MVA, cm^2^	2.93 [2.55–3.11]

Values are mean ± SD, *n* (%), or median [interquartile range]

*C-sept* distance from the coaptation point to the septum, *EF* ejection fraction, *IVST* interventricular septal thickness, *LVOT* left ventricular outflow tract, *MR* mitral regurgitation, *MVA* mitral valve area

The distance between the tip of the AML and the center of the posterior mitral annulus was also measured during diastole. This was recorded as the length of the floating stitch in the present study.

### Mitral valve area

Using spectral continuous color Doppler traces of the diastolic trans-mitral flow, MVA was estimated in the apical four-chamber view [4]. The PHT, the time interval between the maximum early diastolic pressure gradient and the point where the gradient was half the maximum value, was obtained. MVA was calculated as 220 divided by PHT using the PHT method. A minimum of five beats was analyzed for each patient.

This PHT method is an empirically determined formula in rheumatic mitral stenosis and does not provide an accurate MVA. It has been reported that, in sinus rhythm, the PHT method produces values close to those of the 2D-planimetry method. In the present study, the MVA estimated by the PHT method was used [5].

### Surgical technique

**Fig. 1:**
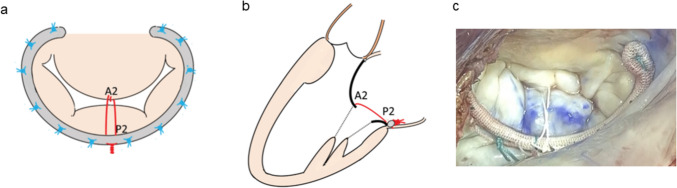
Surgical technique of a floating stitch. **a** Schematic surgical view showing a GORE-TEX suture placed in figure-of-eight fashion at the middle portion of the anterior mitral leaflet (A2) and secured to the posterior annulus (P2). **b** Schematic long-axis view illustrating the mechanism of SAM prevention. **c** Intraoperative photograph showing the application of the floating stitch technique

Under standard cardiopulmonary bypass, septal myectomy was performed using a needle-stick technique. Three 21-gauge needles were inserted from beneath the aortic valve annulus through the hypertrophied septum into the posterior LV wall, marking resection boundaries (width, length, thickness). The needles stabilize the septum and improve exposure by anterior retraction, enabling en bloc resection of consistent thickness [6]. When necessary, abnormalities in the chordae and papillary muscles associated with the anterior mitral leaflet (AML) were addressed. To repair the mitral valve, a partial rigid ring was applied in seven cases, and artificial neo-chordae using GORE-TEX sutures (WL Gore and Associates, Inc., Flagstaff, AZ, USA) were used in two cases. For the remaining three patients with mild MR, the floating stitch was secured to the middle portion of the posterior annulus using pledgets without ring annuloplasty. After the mitral valve repair, the ‘floating stitch’ was applied to the AML. A double-arm GORE-TEX suture (CV4) was applied just to the middle of the tip (A2) of the AML in a figure-of-eight fashion, and both arms of the suture were fixed to the annuloplasty ring at the middle of the posterior mitral annulus (P2) (Fig. 1). The length between A2 and P2 was measured in the systolic phase by transesophageal echocardiography before cardiopulmonary bypass. The final suture length was set as this measured distance plus 2–3 mm, confirmed by a water test. Importantly, this length was maintained shorter than the distance between the posterior mitral annulus and interventricular septum, allowing physiological movement of the AML while preventing contact with the septum. Although rigid rings are generally considered a risk factor for SAM, the floating stitch technique effectively prevents SAM by suspending the anterior mitral leaflet toward the left atrium, thus allowing safe use of partial rigid rings while maintaining adequate valve orifice area.

### Data collection and follow-up

Clinical characteristics, surgical technique, perioperative course, and echocardiographic findings at discharge were obtained from clinical records. Ten patients were followed up by transthoracic echocardiography in our outpatient clinic. A postoperative echocardiogram was performed at discharge and then every year thereafter. The median postoperative observation period was 3.5 (range 1.2–6.6) years. Recent information on survival and re-operation was available for all patients.

### Outcomes

The primary outcome was the durability of the floating stitch technique. A secondary outcome was the hemodynamics of the floating stitch, particularly the progression of mitral stenosis.

### Statistical analysis

There were no missing data in the present study.

Continuous variables are reported as mean ± standard deviation or median and interquartile range (IQR: P25, P75) values and examined with Student’s *t*-test or the Wilcoxon rank-sum test. Preoperative and postoperative echocardiographic parameters were compared using the Wilcoxon signed-rank test. All p-values were two-sided, and p values ≤ 0.05 were considered significant. All statistical analyses were performed using the JMP version 17 software package (SAS Institute, Inc., Cary, NC, USA).

## Results

### Durability of the floating stitch technique

**Fig. 2:**
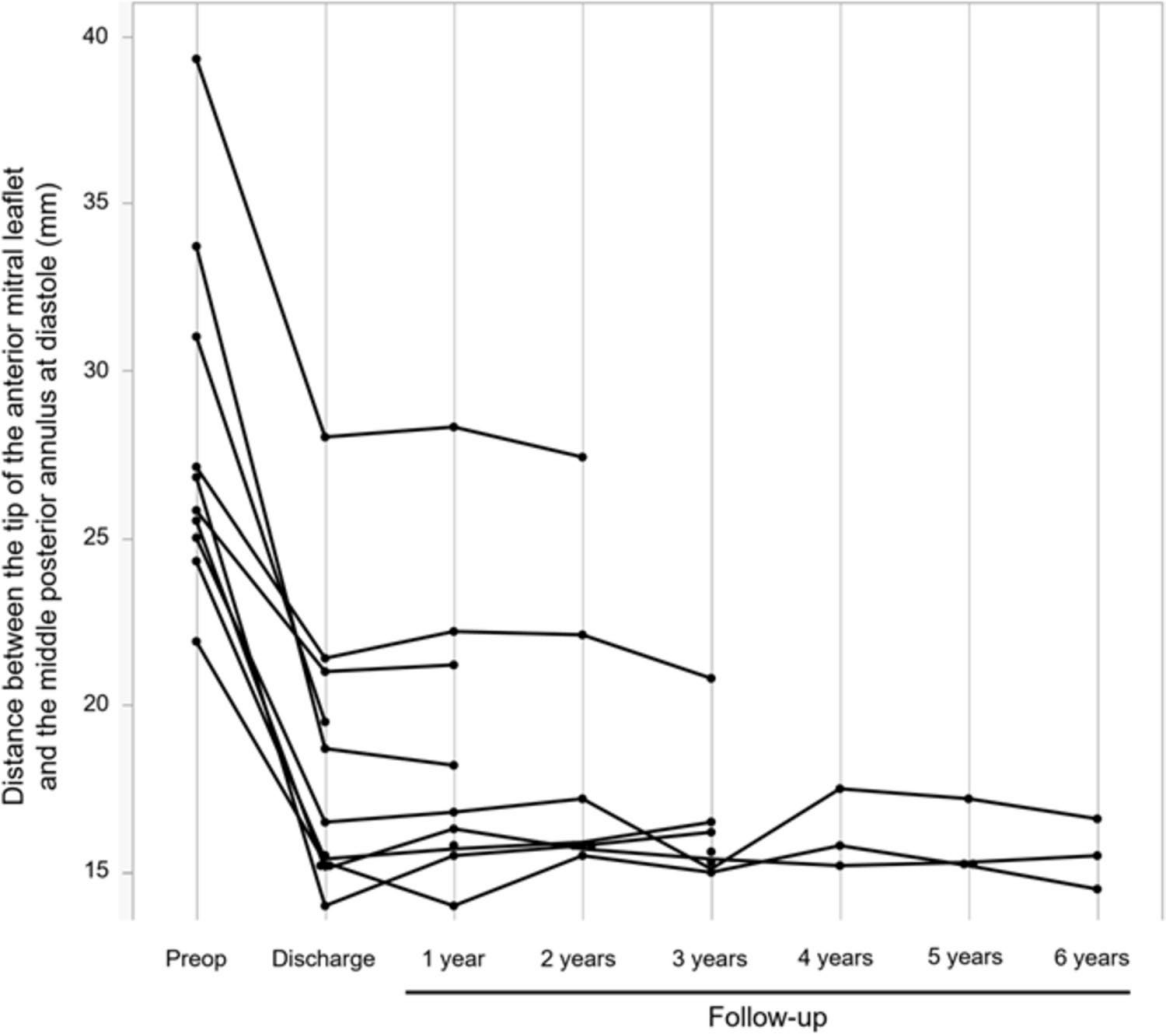
Changes in floating stitch length. Distance between A2 tip and P2 during diastole at preoperative, discharge, and follow-up examinations. Postoperative floating stitch length remains constant. Preop: preoperative

The distance between the tip of the AML (A2) and the middle posterior annulus (P2) during diastole on preoperative and postoperative echocardiographic examinations is shown in Fig. 2. This distance is equivalent to the distance of the floating stitch in the postoperative period. The length of the floating stitch was generally constant, and there were no cases of a broken stitch or of elongation during the follow-up period of up to 6 years. In seven cases in which the floating stitch length was clearly documented in the operative records, this intraoperative measurement was compared with the postoperative echocardiographic measurements, shown in Online Resource 2. These data demonstrate a general concordance between the intended surgical stitch length and the actual postoperative anatomical measurements.

### Changes in mitral valve area and mitral valve function

**Fig. 3:**
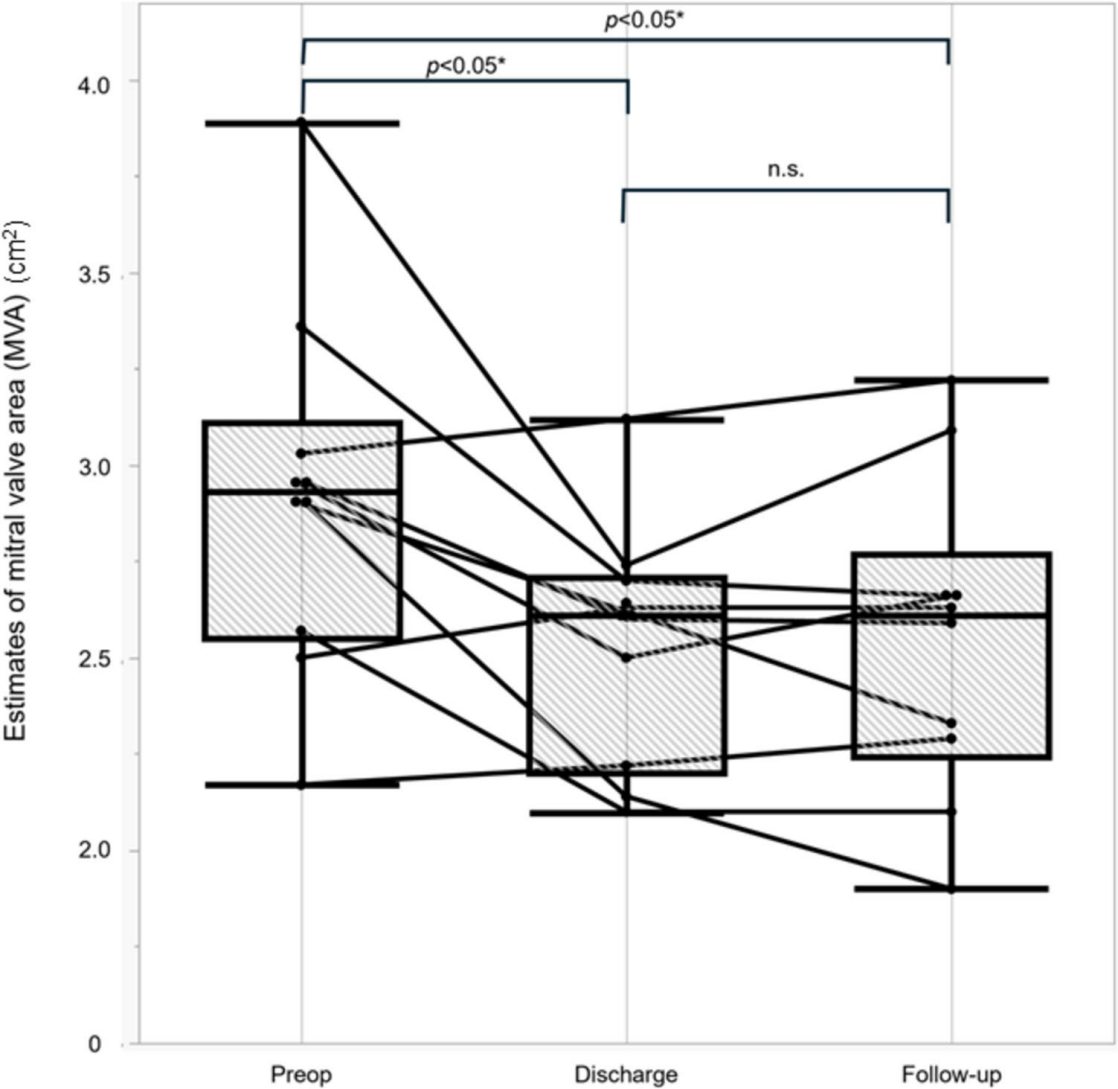
Changes in mitral valve area (MVA). Mitral valve area (MVA) at preoperative, discharge, and latest follow-up examinations is shown. The MVA is decreased about 10% from preoperative to postoperative, but there is no progression of mitral stenosis during the follow-up period. Box plots show median and IQR values. The *p* values are derived from the Wilcoxon signed-rank test. The median MVA is 2.9 cm^2^ (2.6–3.1 cm^2^) before surgery, 2.6 cm^2^ (IQR 2.2–2.7 cm^2^) at discharge, and 2.6 cm^2^ (IQR 2.2–2.8 cm^2^) at the latest follow-up. Preop: preoperative

Estimates of MVA at preoperative, discharge, and the latest follow-up examinations are shown in Fig. 3. The median MVA was 2.9 (IQR 2.6–3.1) cm^2^ before operation, and it decreased to 2.6 (IQR 2.2–2.7) cm^2^ at discharge. MVA showed a significant reduction of about 10% (*p* = 0.02), but sufficient MVA was secured with the floating stitch even in the case with the lowest MVA of 1.9 cm^2^. Though this patient could be classified as having mild mitral valve stenosis, a progressive MVA decrease was not observed during the follow-up period, and at the final follow-up, the patient was NYHA class 1 without signs of heart failure. There were no cases with symptomatic mitral stenosis after the floating stitch procedure. MR had improved in nine patients (90%) to grade 0 or 1 at discharge, and grade 0 or 1 was seen in seven cases, with grade 2 in three cases at the latest follow-up. Owing to the floating stitch, there were no cases of postoperative SAM.

### Left ventricular outflow tract pressure gradient

**Fig. 4:**
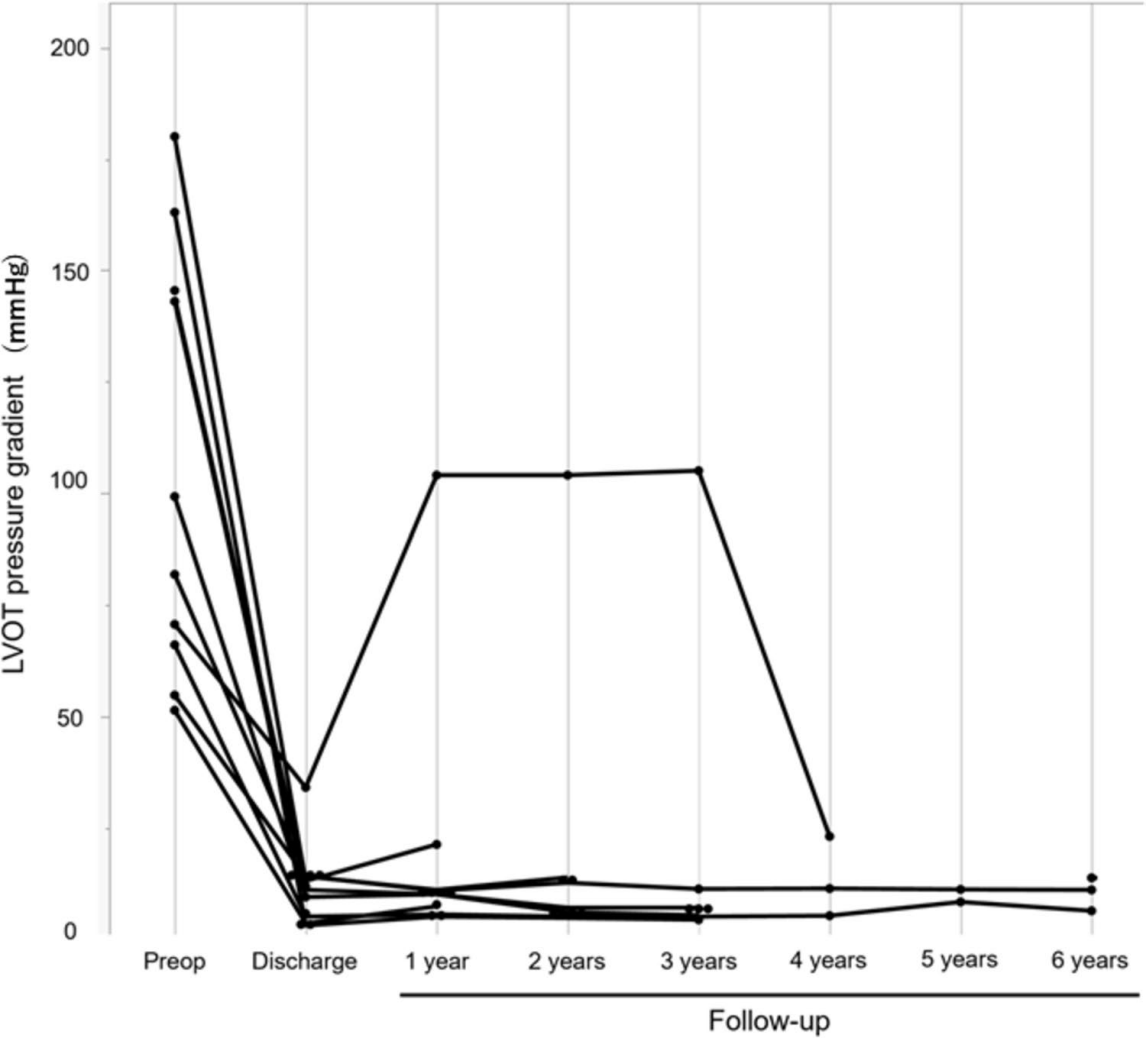
Changes in LVOT pressure gradient. The LVOT pressure gradient at preoperative, discharge, and latest follow-up examinations is shown. In one case, the LVOT pressure gradient increases 1 year after surgery due to recurrent obstruction at the mid-cardiac position; therefore, the patient underwent re-operation with septal myectomy by the apical approach 3 years postoperatively. The postoperative pressure gradient is low and stable in all cases. Preop: preoperative

Figure 4 shows the maximal LVOT pressure gradient preoperatively, postoperatively, and during follow-up. At the time of discharge, all cases showed a significant decrease in the LVOT pressure gradient (median: 12 mmHg, IQR: 4.6–14 mmHg). However, one case showed an increasing pressure gradient at the mid-cardiac position between the papillary muscles and the interventricular septum, but there was neither SAM nor a pressure gradient under the subaortic valve area. The patient underwent re-operation with septal myectomy via the apical approach 3 years after the initial surgery, and the LVOT pressure gradient again decreased significantly. The LVOT pressure gradient remained stable at low levels during the follow-up period in all other cases.

### Other hemodynamic parameters

**Fig. 5:**
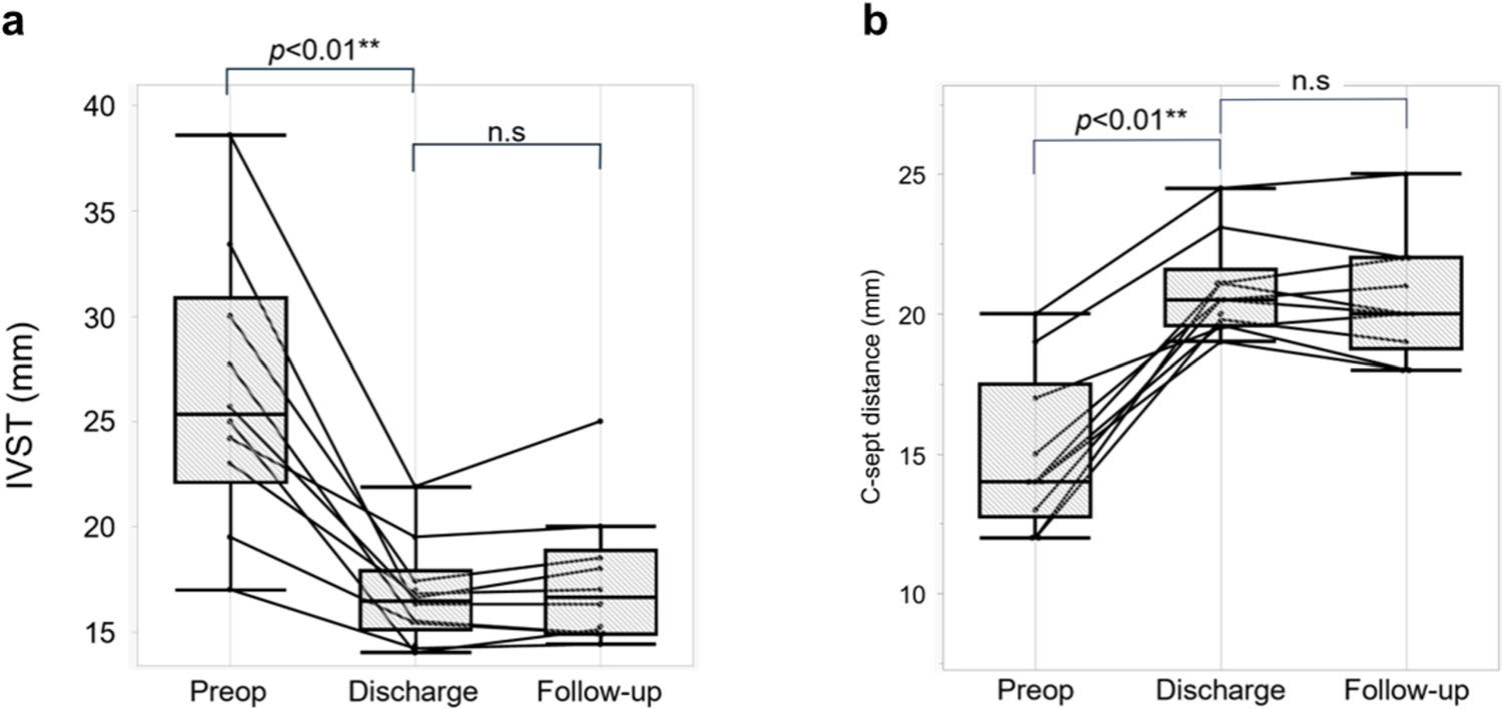
Changes in IVS thickness and C-sept distance. **a** Interventricular septal (IVS) thickness is significantly decreased (*p* < 0.01**) postoperatively compared with preoperatively. **b** The coaptation point to septum (C-sept) distance is significantly increased (*p* < 0.01**) postoperatively compared with preoperatively

IVST was significantly decreased (*p* < 0.01), and C-sept distance was significantly increased (*p* < 0.01) postoperatively compared with preoperatively (Fig. 5). Both IVST and C-sept distance showed stable values during the follow-up period. Though C-sept distance was less than 20 mm in some cases, which is generally considered a risk factor for SAM, there were no recurrences of SAM due to the floating stitch. Online Resource 1 shows the echocardiographic changes in C-sept distance and the effectiveness of the floating stitch in preventing SAM across the cardiac cycle.

### Survival, re-operation, and postoperative NYHA class

All patients survived the observation period. One case required re-operation 3 years after the initial surgery due to mid-cardiac obstruction. All patients were in NYHA class 1 at the latest follow-up.

## Discussion

Subaortic septal myectomy is the gold standard for relieving LVOT obstruction and reducing associated SAM and MR [7, 8]. Septal myectomy redirects ejection flow anteriorly and medially away from the AML, and this physiological flow reduces the dragging force on the AML. However, direct surgical management for SAM is another key aspect of surgery for HOCM.

### Advantages of the floating stitch

The floating stitch we have developed is an easy and simple technique to relieve SAM. Functional mitral stenosis is the only concern with this technique, but MVA can be sufficiently restored with postoperative MVA of 2.6 (IQR 2.2–2.7) cm^2^. The floating stitch suspends the tip of the AML, and it mildly restricts opening of the center of the AML, but there is no restriction of movement of both sides of the AML. In addition, it does not interfere with posterior mitral leaflet (PML) movement. For these reasons, we believe that the floating stitch can secure sufficient MVA. The physical restriction of anterior movement of the AML during systole virtually eliminates the possibility of SAM.

We have routinely used CV4 sutures for the floating stitch. They are GORE-TEX sutures, which are usually used as neo-chordae for mitral valve repair, and their excellent durability has been proven. The floating stitch receives the left atrial pressure during diastole, but it never receives left ventricular pressure during systole. The durability of the floating stitch is not an issue, because it receives only atrial pressure. In fact, no cases developed cutting or elongation of a floating stitch during the follow-up period of up to 6 years.

A similarly simple approach for SAM treatment, edge-to-edge repair by Alfieri et al., has shown good results in SAM management, with Myers et al. reporting complete elimination of SAM in 65 patients and only one recurrence during follow-up [9]. However, edge-to-edge sutures may increase the risk of mitral stenosis, especially in patients with a small mitral annulus. It has been reported that, after percutaneous edge-to-edge mitral valve repair, the mitral valve orifice area may be reduced by up to 60% [10]. Unlike edge-to-edge repair that sutures both leaflets together, creating a double orifice with significant reduction in valve area, the floating stitch suspends only the anterior leaflet while maintaining substantial leaflet mobility. By preserving complete posterior leaflet function, this technique limits valve area reduction to approximately 10%. In addition, whereas the edge-to-edge suture is subjected to systolic pressure, the floating stitch is only subjected to diastolic pressure, which may have led to better long-term durability and hemodynamic outcomes in the present series.

### Systolic anterior motion-suppression effect of the floating stitch

The C-sept distance was significantly higher postoperatively than preoperatively. This was due to sufficient myectomy and relieving SAM by the floating stitch. C-sept distance remained constant during follow-up. This may suggest that the SAM-suppression effect of the floating stitch is constant.

One of the patients in the present study had recurrent obstruction. The preoperative LVOT peak pressure gradient was 51 mmHg, and grade 2 MR had occurred due to SAM. After septal myectomy and management of SAM with a floating stitch, postoperative echocardiography showed that the pressure gradient decreased to 15 mmHg, but it increased to 102 mmHg 1 year later due to recurrent obstruction at the mid-cardiac position between the papillary muscles and the IVS. Notably, SAM did not recur despite a stronger pressure gradient. This finding suggests the remarkable efficacy of the floating stitch in preventing SAM.

### Use of the floating stitch for SAM in mitral valve repair and the minimally invasive cardiac surgery procedure

As we have reported previously, the floating stitch is also useful in the prevention and treatment of SAM after mitral valve repair [11]. When SAM does not resolve despite medical therapy, surgical intervention with aortic re-clamping is required.

In addition, in recent years, the use of minimally invasive cardiac surgery (MICS) has been increasing not only for mitral valve surgery, but even for HOCM surgery. Wei et al. reported the usefulness of the MICS procedure for septal myectomy, highlighting its excellent exposure and effectiveness in relieving obstruction [12]. Therefore, a technique that can be easily performed even in MICS is required.

With respect to these two points, the floating stitch technique is simple and easy, and it can be applied without direct manipulation to the valve or sub-valvular apparatus.

### Study limitations

This study was limited by its single-center, retrospective design with only 10 HOCM patients. This study also was limited in demonstrating the effectiveness of the floating stitch because there was no comparison with control cases without the floating stitch. Further studies with larger patient cohorts and extended follow-up periods are needed to definitively establish the safety, durability, and universal applicability of the floating stitch technique.

## Conclusion

The floating stitch technique showed excellent SAM suppression and durability. MVA decreased about 10% with the floating stitch, but sufficient MVA was achieved without functional mitral stenosis. The combination of septal myectomy and floating stitch technique is a simple and reproducible procedure for HOCM, especially for cases with severe SAM.

## Supplementary Information

Below is the link to the electronic supplementary material.

Supplementary file1 (PDF 411 KB)

Supplementary file2 (PDF 73 KB)

## Data Availability

Data are available from the corresponding author upon reasonable request.
