# Supplementary material for: Mid-term results of the floating stitch for systolic anterior motion in hypertrophic obstructive cardiomyopathy
Source: Gen Thorac Cardiovasc Surg. 2025 Jun 12;73(12):885–92. doi: 10.1007/s11748-025-02167-6 (PMC12681494; doi:10.1007/s11748-025-02167-6)
Supplement: Supplementary file 1 — Supplementary file1 (PDF 411 KB) [file 11748_2025_2167_MOESM1_ESM.pdf]

Online Resource 1

Mid-term results of the floating stitch for systolic anterior motion in hypertrophic obstructive cardiomyopathy

General Thoracic and Cardiovascular Surgery

Tomonari Uemura, MD[1], Akihiko Usui, MD, PhD[2], Yoshiyuki Tokuda, MD, PhD[1], Yuji Narita, MD, PhD[1], Masato Mutsuga, MD, PhD[1]

[1]Department of Cardiac Surgery, Nagoya University Graduate School of Medicine, Nagoya, Japan

[2]Department of Cardiovascular Surgery, Fujita Health University Okazaki Medical Center, Okazaki, Japan

Corresponding Author: Dr. Tomonari Uemura (uemura.tomonari1988@gmail.com)

Effects of the floating stitch and changes in C-sept distance in the preoperative and postoperative phases

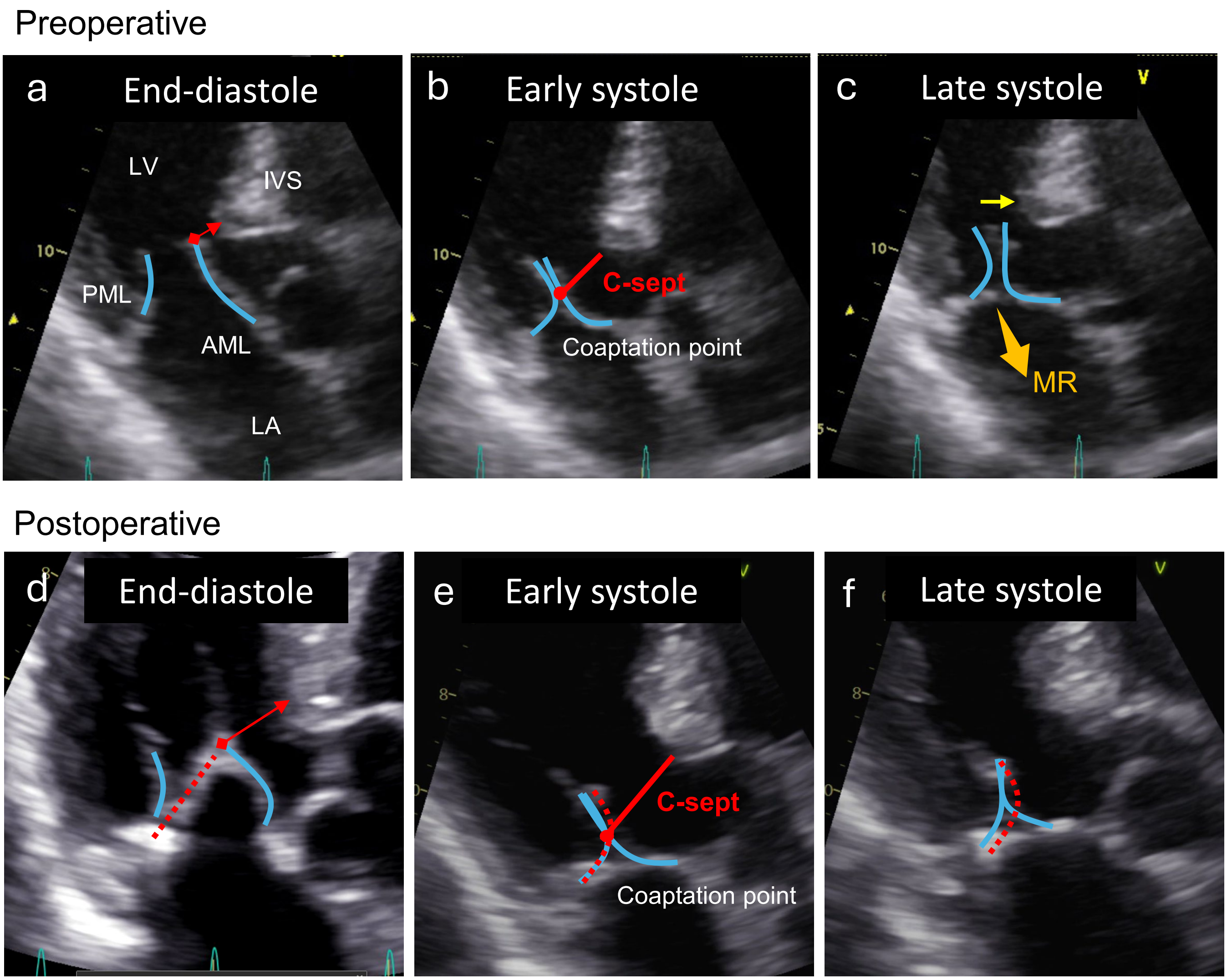

a. In the preoperative end-diastolic phase, the anterior mitral leaflet (AML) is in close proximity to the interventricular septum (IVS); the red arrow indicates the distance between the AML tip and the IVS.

b. In early systole, the coaptation point is displaced anteriorly toward the IVS, resulting in a shortened C-sept distance.

c. Systolic anterior motion (SAM) occurs (yellow arrows), leading to mitral regurgitation (MR).

d. After septal myectomy and placement of the floating stitch (indicated by the red dotted line), the AML is consistently pulled toward the left atrium during diastole, maintaining a sufficient distance from the IVS (red arrow).

e. In early systole, the C-sept distance is preserved due to the combined effect of septal myectomy and the floating stitch.

f. SAM is not observed.
