# Supplementary material for: Mid-term results of the floating stitch for systolic anterior motion in hypertrophic obstructive cardiomyopathy
Source: Gen Thorac Cardiovasc Surg. 2025 Jun 12;73(12):885–92. doi: 10.1007/s11748-025-02167-6 (PMC12681494; doi:10.1007/s11748-025-02167-6)
Supplement: Supplementary file 2 — Supplementary file2 (PDF 73 KB) [file 11748_2025_2167_MOESM2_ESM.pdf]

Online Resource 2

Mid-term results of the floating stitch for systolic anterior motion in hypertrophic obstructive cardiomyopathy

General Thoracic and Cardiovascular Surgery

Tomonari Uemura, MD[1], Akihiko Usui, MD, PhD[2], Yoshiyuki Tokuda, MD, PhD[1], Yuji Narita, MD, PhD[1], Masato Mutsuga, MD, PhD[1]

[1]Department of Cardiac Surgery, Nagoya University Graduate School of Medicine, Nagoya, Japan

[2]Department of Cardiovascular Surgery, Fujita Health University Okazaki Medical Center, Okazaki, Japan

Corresponding Author: Dr. Tomonari Uemura (uemura.tomonari1988@gmail.com)

Comparison of Intraoperative Floating Stitch Length and Corresponding Postoperative Echocardiographic Measurements

Distance between the tip of the AML and the middle posterior annulus at diastole on postoperative TTE (mm)

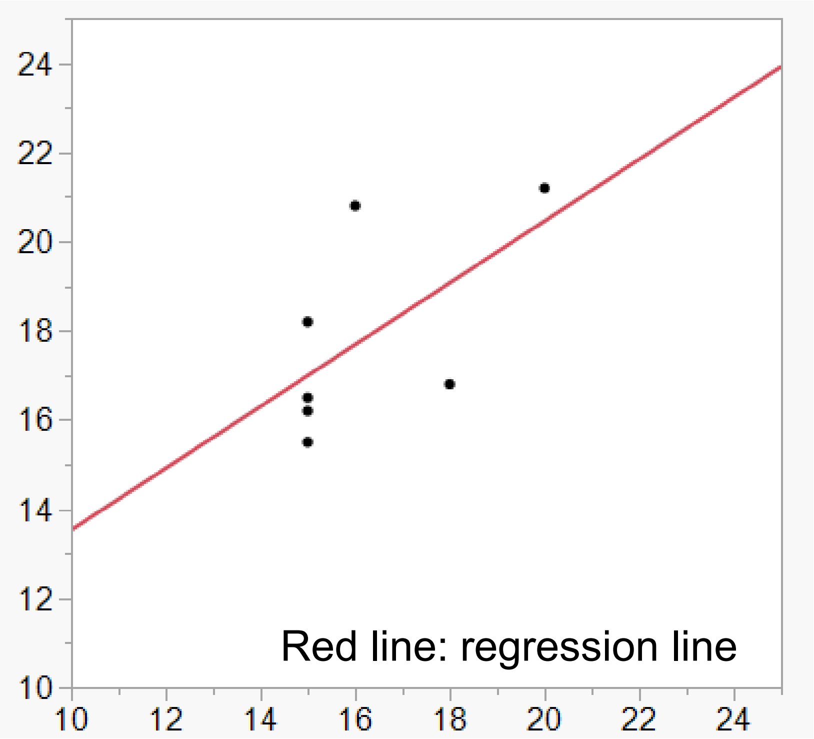

Floating stitch length during the operation (mm)

AML: anterior mitral leaflet
TTE: transthoracic echocardiography
